# Identifying the Prognostic Factors Affecting the Conversion From Laparoscopic Cholecystectomy to Open Cholecystectomy in Acute Cholecystitis

**DOI:** 10.7759/cureus.73975

**Published:** 2024-11-19

**Authors:** Dhanalakshmi Kadirvel, Sreedevi Vasudevan, Sundeep Selvamuthukumaran, Sunidhi Rajput, Pola Govardhan Kumar

**Affiliations:** 1 Department of General Surgery, Sree Balaji Medical College and Hospital, Chennai, IND

**Keywords:** acute calculus cholecystitis, cholecystitis, gallbladder, laparoscopic cholecystectomy, prognostic factor

## Abstract

Introduction

Acute cholecystitis, commonly caused by gallstones, is a prevalent surgical emergency worldwide. Laparoscopic cholecystectomy (LC) is the gold standard for treatment, but the timing is crucial, with early surgery (within seven days) reducing complications. Identifying prognostic factors such as age, sex, white blood cell (WBC) count, C-reactive protein (CRP), and gallbladder wall thickness can help predict outcomes and reduce the need for conversion to open surgery.

Methods

A prospective longitudinal observational study was conducted at Sree Balaji Medical College and Hospital, Chennai, on 60 patients diagnosed with acute cholecystitis. The inclusion criteria involved clinical signs (right upper abdominal tenderness, temperature >37.5°C, WBC >10,000 cells/mm³) and ultrasound findings (gallstones, thickened gallbladder, sonographic Murphy's sign). Data on prognostic factors (WBC, CRP, ultrasound, intraoperative findings) were collected and analyzed using IBM SPSS Statistics for Windows, Version 28.0 (Released 2021; IBM Corp., Armonk, New York, United States), with chi-squared tests to evaluate associations between these factors and surgical outcomes.

Results

Patients aged 18-30 had the highest success rate for early LC, with only 10.5% converting to open surgery, whereas older patients (>50 years) had a higher conversion rate (33.3%). A higher body mass index (BMI) (>30) was linked to increased conversions (26.3%), as was the presence of hypertension (23.1%) and diabetes (22.2%). Elevated WBC and CRP levels were significant predictors of conversion (24.2% and 22.2%, respectively). Intraoperative factors such as gallbladder wall thickening (>4 mm) and pericholecystic fluid were associated with conversion rates of 20.8% and 25%, respectively. Converted cases had longer operative times and extended recovery periods. The predictive model for conversion showed a sensitivity of 88.9% and a specificity of 90%.

Conclusion

Key prognostic factors influencing the success of early LC include age, BMI, comorbidities, and inflammatory markers such as WBC and CRP. Younger patients had higher success rates, whereas older and obese patients were at greater risk of conversion. Preoperative optimization and early surgical intervention are critical for reducing conversion rates and improving outcomes. The predictive model's accuracy provides clinicians with a valuable tool for surgical planning.

## Introduction

Acute cholecystitis, primarily caused by gallstones, is a common surgical emergency worldwide, with 10-15% of the population developing gallstones and 1-3% experiencing acute cholecystitis [1]. The incidence is increasing, particularly in developed nations and in countries like India, where dietary habits are rapidly changing. In India, the prevalence of cholelithiasis ranges from 5% to 10%, with higher rates observed in women.

Laparoscopic cholecystectomy (LC) is the gold standard for treating acute cholecystitis, offering the benefits of minimally invasive surgery, such as shorter recovery times and fewer complications. The timing of the procedure is crucial, with early surgery (within seven days) shown to reduce complications. The Tokyo Guidelines 2013 [2] recommends early cholecystectomy within 72 hours of symptom onset, although the window can be extended to seven days in patients who remain stable and do not have severe inflammation or other contraindications. The Tokyo Guidelines 2018 (TG18) [2] reaffirms the importance of early LC for acute cholecystitis and reinforces the 72-hour window as optimal.

This extension is meant for cases where logistical or clinical constraints prevent surgery within the 72-hour window but where the patient's condition allows for early intervention without significantly increased risk. The preference, however, is always to operate within the initial 72 hours if possible, as studies have shown better outcomes when surgery is performed within this timeframe.

Despite its success, some patients experience complications or require conversion to open surgery. Prognostic factors such as sex, white blood cell (WBC) count, C-reactive protein (CRP), alkaline phosphatase, and amylase levels can help predict surgical outcomes. Elevated CRP and a thickened gallbladder wall are associated with an increased risk of conversion. Identifying these factors can enhance patient selection, reduce complications, and optimize surgical outcomes. This study aims to evaluate the role of these factors in predicting successful early LC.

## Materials and methods

This prospective longitudinal observational study was conducted from March 2023 to June 2024 for a period of 15 months in the Department of General Surgery at Sree Balaji Medical College and Hospital, Chennai, to assess the prognostic factors influencing the likelihood of conversion from laparoscopic to open surgery in patients undergoing early LC for acute cholecystitis. Informed consent was obtained in accordance with the Sree Balaji Medical College and Hospital Institutional Human Ethics Committee (IHEC) guidelines (approval number: 002/SBMCH/IHEC/2023/1952).

Patients were selected for the study based on specific inclusion and exclusion criteria. The inclusion criteria comprised clinical signs such as acute right upper abdominal tenderness, a temperature exceeding 37.5°C at presentation, and a WBC count greater than 10,000 cells/mm³. Ultrasound (USG) findings required the presence of gallstones in a thickened and edematous gallbladder, a positive sonographic Murphy's sign, and pericholecystic fluid collection. Additionally, histopathological confirmation of acute cholecystitis was necessary.

The exclusion criteria included patients with choledocholithiasis detected on USG, those unfit for semi-emergency surgery due to uncontrolled hypertension, diabetes, and hepatic or renal disease, pregnant women, patients presenting with jaundice or cholangitis, and individuals who refused surgery or did not consent to participate. A purposive sampling method was used to select patients. Data collection involved detailed clinical histories, laboratory investigations (WBC count, CRP, serum amylase, alkaline phosphatase), USG assessments, and intraoperative findings such as edematous gallbladder, mucocele, gangrene, or empyema. Postoperative follow-up recorded complications and the length of hospital stay.

A limitation of this study is the reduced sample size; while originally calculated for 90 patients based on a 4% prevalence of acute cholecystitis, only 60 patients were ultimately included due to feasibility constraints within the study period. This smaller sample may have impacted the statistical power of the findings, potentially limiting the detection of subtle yet clinically important differences in success and conversion rates, particularly in subgroup analyses by age, body mass index (BMI), and comorbidities. Additionally, the reduced sample size could affect the generalizability of the results, as a larger, more diverse sample might provide a broader perspective on predictors of conversion. Despite this, statistical analysis was rigorously conducted using IBM SPSS Statistics for Windows, Version 28.0 (Released 2021; IBM Corp., Armonk, New York, United States), applying descriptive statistics and chi-squared tests to evaluate associations between prognostic factors and surgical outcomes. Nonetheless, future studies with larger samples and potentially multicenter designs would be valuable to confirm these results and offer a more comprehensive view of the factors influencing LC success.

## Results

Younger patients (18-30 years) demonstrated the highest success rate for LC at 89.5%, with a conversion rate of only 10.5%. In contrast, older patients (>50 years) exhibited the lowest success rate at 66.7% and the highest conversion rate at 33.3%. Male patients had a success rate of 78.9%, while female patients achieved a higher success rate of 90.9% and a lower conversion rate of 9.1%. Regarding BMI, patients with a BMI of less than 25 had an 87% success rate and a 13% conversion rate, whereas those with a BMI over 30 had the lowest success rate at 73.7% and the highest conversion rate of 26.3%. This increase indicates that higher BMI is associated with more complex surgical challenges, such as reduced intra-abdominal visibility and limited instrument maneuverability, both of which can necessitate conversion to open surgery. Comorbidities also significantly affected outcomes, with patients having none experiencing the highest success rate of 86.8% and the lowest conversion rate of 13.2%. Additionally, laboratory parameters influenced surgical outcomes; patients with WBC counts greater than 10,000/mm³ had a success rate of 75.8%, and those with alkaline phosphatase levels exceeding 147 IU/L had a lower success rate of 72% (Table 1).

**Table 1:** Patient characteristics, comorbidities, and laboratory parameters influencing the success and conversion rates of early LC BMI: body mass index; LC: laparoscopic cholecystectomy; WBC: white blood cells; CRP: C-reactive protein

Parameters	Total n (%)	Successful LC n (%)	Conversion rate n (%)
Age group (years)			
18-30	19 (31.7%)	17 (89.5%)	2 (10.5%)
31-50	32 (53.3%)	26 (81.3%)	6 (18.8%)
>50	9 (15%)	6 (66.7%)	3 (33.3%)
Gender			
Male	38 (63.3%)	30 (78.9%)	8 (21.1%)
Female	22 (36.7%)	20 (90.9%)	2 (9.1%)
BMI range (kg/m²)			
<25	23 (38.3%)	20 (87%)	3 (13%)
25-30	18 (30%)	16 (88.9%)	2 (11.1%)
>30	19 (31.7%)	14 (73.7%)	5 (26.3%)
Comorbidity			
Hypertension	13 (21.7%)	10 (76.9%)	3 (23.1%)
Diabetes	9 (15%)	7 (77.8%)	2 (22.2%)
None	38 (63.3%)	33 (86.8%)	5 (13.2%)
Laboratory parameter			
WBC count >10,000/mm³	33 (55%)	25 (75.8%)	8 (24.2%)
CRP >10 mg/L	27 (45%)	21 (77.8%)	6 (22.2%)
Alkaline phosphatase >147 IU/L	25 (41.7%)	18 (72%)	7 (28%)
Serum amylase >85 IU/L	18 (30%)	14 (77.8%)	4 (22.2%)

Gallbladder wall thickness was examined at intervals such as >4 mm and >8 mm. We observed a clear gradient where greater wall thickness correlated with progressively higher conversion rates, suggesting that increased inflammation or fibrosis adds to surgical complexity, thus suggesting that patients with >8-mm-thick gallbladder wall have a lower LC success rate and a higher likelihood of requiring conversion compared to those with thicknesses between 4 mm and 8 mm. Similarly, WBC counts were compared at >10,000 cells/mm³ and >20,000 cells/mm³, showing that very high WBC levels are associated with severe inflammatory response or infection, significantly increasing the likelihood of conversion to open surgery. In the same way, CRP levels of more than 250 mg/L showed that extremely high CRP is predictive of conversion, pointing to a level of inflammation that makes laparoscopic access more challenging compared to 150-200 mg/L.

Patients with a gallbladder wall thickness greater than 4 mm had a 79.2% success rate for LC and a 20.8% conversion rate. Those with a gangrenous gallbladder had an 83.3% success rate, with a conversion rate of 16.7%. Patients presenting with a mucocele had a success rate of 85.7% and a conversion rate of 14.3%. Additionally, patients with pericholecystic fluid showed a 75% success rate and a 25% conversion rate. Postoperative complications included bile duct injury in two patients (3.3%), with an equal distribution of outcomes: 50% success in the LC group and 50% requiring conversion to open surgery. Surgical site infections were observed in six patients (10%), of whom 66.7% underwent successful LC, while 33.3% needed conversion. Intra-abdominal abscesses occurred in four patients (6.7%), with a 50% success rate for LC and a 50% conversion rate (Table 2).

**Table 2:** Intraoperative findings and postoperative complications LC: laparoscopic cholecystectomy

Finding	Total n (%)	Successful LC n (%)	Conversion rate n (%)
Gallbladder wall thickness >4 mm	24 (40%)	19 (79.2%)	5 (20.8%)
Gangrenous gallbladder	12 (20%)	10 (83.3%)	2 (16.7%)
Mucocele	7 (11.7%)	6 (85.7%)	1 (14.3%)
Pericholecystic fluid	16 (26.7%)	12 (75%)	4 (25%)
Complication			
Bile duct injury	2 (3.3%)	1 (50%)	1 (50%)
Surgical site infection	6 (10%)	4 (66.7%)	2 (33.3%)
Intra-abdominal abscess	4 (6.7%)	2 (50%)	2 (50%)

The mean duration of surgery for successful LC was 72±14 minutes, while it was significantly longer at 115±18 minutes for conversions. Postoperative stay averaged 2.1±0.4 days for successful LC compared to 4.8±0.7 days for converted cases (Table 3).

**Table 3:** Surgery and postoperative stay LC: laparoscopic cholecystectomy; SD: standard deviation

Parameter	Successful LC (mean±SD)	Conversion (mean±SD)
Duration of surgery	72±14 minutes	115±18 minutes
Postoperative stay (days)	2.1±0.4 days	4.8±0.7 days

A strong correlation (0.76) between BMI and duration of surgery suggests that higher BMI leads to longer surgery times. Similarly, WBC count is moderately correlated (0.72) with surgery duration, indicating that higher inflammation can make surgeries more complex (Table 4).

**Table 4:** Correlation matrix BMI: body mass index

Variable	BMI	Duration of surgery	White blood cell count
BMI	1.00	0.76	0.65
Duration of surgery (minutes)	0.76	1.00	0.72
White blood cell count	0.65	0.72	1.00

The model's sensitivity is 88.9%, indicating high accuracy in identifying patients who needed conversion, while specificity (90%) shows strong performance in identifying patients who successfully underwent laparoscopic surgery (Table 5).

**Table 5:** Sensitivity and specificity

True positive	False positive	True negative	False negative	Sensitivity (%)	Specificity (%)
8	2	18	1	88.9%	90%

## Discussion

In this study, younger patients (18-30 years) had the highest success rate for early LC, with only 10.5% converting to open surgery, whereas older patients (>50 years) exhibited a significantly higher conversion rate (33.3%). This finding aligns with Janjic et al. [3], who reported that age-related anatomical changes and increased tissue fibrosis in older patients elevate the likelihood of conversion. Similarly, male patients had a higher conversion rate (21.1%) compared to females (9.1%), which can be attributed to more challenging anatomical factors in males, as observed in this study and consistent with findings by Al-Mulhim [4].

Regarding the BMI distribution, patients with a BMI >30 had a higher conversion rate (26.3%), consistent with findings by Rosen et al. [5], who emphasized that obesity complicates visualization during laparoscopic surgery, leading to longer operative times and an increased conversion rate. The positive correlation between BMI and surgery duration (0.76) further supports the increased complexity in obese patients, as noted in previous studies.

In terms of comorbidities, patients with hypertension and diabetes had higher conversion rates (23.1% and 22.2%, respectively). This is similar to the findings of Gul et al. [6], indicating that preoperative optimization of these conditions can improve surgical outcomes. Elevated rates of inflammation and vascular changes in hypertensive and diabetic patients can complicate surgery by causing changes in tissue structure, blood flow, and healing capacity. Studies have shown that these comorbidities are associated with increased surgical risk, including higher conversion rates, prolonged operative times, and longer hospital stays.

Additionally, elevated WBC counts and CRP levels were associated with higher conversion rates (24.2% and 22.2%, respectively), in line with Sert et al. [7], who identified inflammatory markers, especially CRP, as strong predictors of conversion in cases of acute cholecystitis.

Intraoperative findings such as gallbladder wall thickening (>4 mm) and pericholecystic fluid were also significant predictors of conversion (20.8% and 25%, respectively). These results are comparable to those reported by Bundgaard et al. [8], who found that severe gallbladder inflammation significantly increases the need for conversion.

The data on surgical duration and postoperative stay show that conversion cases had significantly longer surgeries (115±18 minutes) and extended postoperative recovery periods (4.8±0.7 days). This is consistent with the findings of Gutt et al. [9], who highlighted that conversion to open surgery prolongs recovery. Postoperative complications such as bile duct injuries occurred in two patients (3.3%), split evenly between those who remained in LC (50%) and those who converted to open surgery (50%). Surgical site infections affected six patients (10%), with four (66.7%) completing LC successfully and two (33.3%) requiring conversion. Intra-abdominal abscesses were noted in four patients (6.7%), with a 50% rate of conversion. Additionally, the bleeding rate was found to be 2% in LC and 6% in open surgery cases, as the minimally invasive approach generally involves less tissue trauma and a lower risk of vascular injury. Similarly, for bowel injuries, it was found to be 1% in LC and 4% in open surgery cases, as open surgery can increase the risk of incidental bowel handling and injury, corroborating the incidence of such complications as reported by Rosen et al. [5].

A limitation of this study is the reduced sample size; while originally calculated for 90 patients based on a 4% prevalence of acute cholecystitis, only 60 patients were ultimately included due to feasibility constraints within the study period. This reduced sample size may have impacted the statistical power of the study, potentially limiting the ability to detect smaller or more nuanced differences in outcomes, especially in subgroup analyses by age, BMI, and comorbidities. Additionally, a smaller sample may restrict generalizability, as a larger sample could provide more robust insights into conversion rates and outcomes across varied patient demographics and clinical presentations. Despite these limitations, the study still demonstrated significant correlations and provided valuable insights, though larger follow-up studies could confirm these findings in a broader patient population.

Conversion to open surgery occurred at rates of 33.3% in patients over 50 years and 26.3% in those with a BMI over 30 and in patients with comorbidities such as hypertension (23.1%) and diabetes (22.2%). The small sample size as mentioned above may have introduced a bias in conversion rates, especially in subgroups like elderly or obese patients where conversion is inherently more likely. 

Finally, the sensitivity (88.9%) and specificity (90%) of the predictive model underscore the model's reliability in identifying patients at risk for conversion and in aiding clinical decision-making. These comparisons with existing literature confirm the relevance and applicability of this study's findings to the broader field of LC.

## Conclusions

This study highlights the critical prognostic factors influencing the success of early LC in acute cholecystitis. Age, BMI, comorbidities, and inflammatory markers such as WBC and CRP significantly impact conversion rates. Younger patients had a higher success rate with LC, while older patients were more prone to conversion. A higher BMI increases the surgical complexity and the risk of conversion, emphasizing the need for specialized care in obese patients. Preoperative management of comorbidities and early intervention, particularly before the onset of severe inflammation, are key to reducing conversion rates. The predictive model's high sensitivity and specificity confirm the importance of these factors in surgical planning, providing clinicians with a reliable guide for improving patient outcomes.
